# Cutaneous Mucormycosis in an Immunocompetent Child following a Minor Skin Trauma

**DOI:** 10.1155/2022/7005024

**Published:** 2022-03-22

**Authors:** Rashmi Shingde, Rebecca Cui, Ruwan Perera

**Affiliations:** Department of General Surgery, Dubbo Base Hospital, Dubbo, NSW 2830, Australia

## Abstract

Cutaneous mucormycosis is a rare infection by the *Zygomycetes* class of fungi, which carries significant morbidity and mortality. While typically associated in patients with underlying immunocompromise (especially in the current era of COVID-19), it may also be seen in immunocompetent patients. We report a case of a healthy 4-year-old girl with acute right leg cellulitis and abscess formation, who required surgical debridement following poor response to antibiotic therapy and initial incision and drainage. Tissue histopathology returned cutaneous zygomycosis despite negative tissue cultures. At four-week follow-up, her wound was healed well. Clinicians should maintain a high degree of clinical suspicion for cutaneous mucormycosis given its potential for rapidly progressive and disseminated disease. Currently, the mainstay of diagnostic investigations is tissue histopathology, with a growing role for tissue fungal PCR. Treatment involves multidisciplinary management between surgeons and Infectious Diseases team to guide the role for surgical debridement and antifungal therapy.

## 1. Introduction

Cutaneous mucormycosis is a rare infection caused by the *Zygomycetes* class of fungi, normally found in soil and decaying vegetation [1]. Other than cutaneous infection (22%), mucormycosis can present as rhino-orbito-cerebral (34%), pulmonary (20%), gastrointestinal infection (8%), or disseminated disease (13%) [2]. Cutaneous mucormycosis is associated with significant mortality (up to 32% [2]); thus, early diagnosis and prompt therapy remain paramount. This case report outlines key clinical practice points for the diagnosis and management of cutaneous mucormycosis.

## 2. Case Report

A 4-year-old Caucasian girl presented to a regional hospital with a three-day history of the right leg cellulitis, after sustaining a minor splinter from burr grass while playing in a paddock. She was an otherwise healthy child with no relevant medical history. On examination, she was febrile but hemodynamically stable, with a localised 3 × 3 cm area of cellulitis over the distal anterior surface of her right leg. There were erythema and induration, but no obvious fluctuance. On investigation, white cell count was 17.6 × 10^9^/L with predominant neutrophilia and lymphocytosis, and C-reactive protein was 23 mg/L. The right leg X-ray was unremarkable, and ultrasound found a 31 × 12 × 28 mm area of subcutaneous oedema, with no drainable collection.

**Figure 1:**
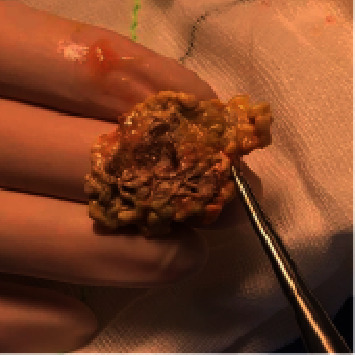
Debrided mass from the right leg.

**Figure 2:**
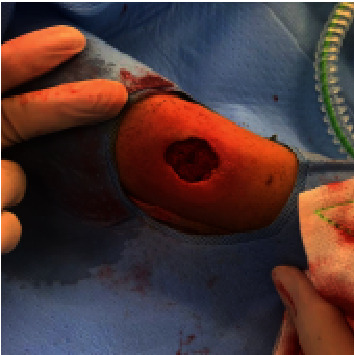
Debridement to the level of the muscle.

She was commenced on intravenous cefotaxime, flucloxacillin, and vancomycin. Despite antibiotics, her cellulitis progressed, and a fluctuant collection developed. On day 2, she underwent an incision and drainage of the abscess where purulent fluid was expressed, and the wound was packed. Postoperatively, she had persistent low-grade fevers and ongoing purulent discharge. Thus, on day 3, she underwent a further debridement and washout of the wound. Intraoperative findings included a sloughy, devitalised tissue mass (Figure 1) which was debrided to healthy muscle (Figure 2). Preliminary tissue cultures showed no growth, and she was discharged on one week of oral amoxicillin/clavulanate with daily dressing changes.

**Figure 3:**
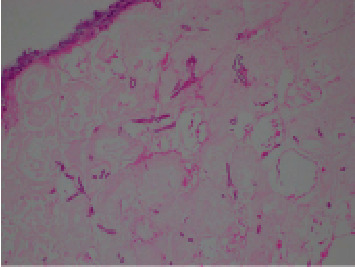
Tissue biopsy showing broad pauciseptate fungal hyphae with wide-angle branching in necrotic adipose tissue, consistent with zygomycosis (H&E, ×200).

**Figure 4:**
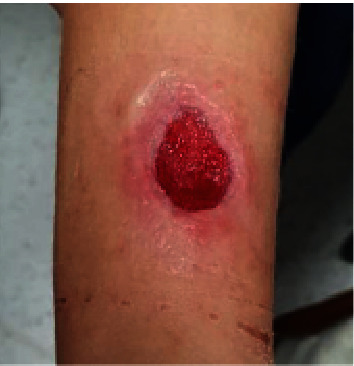
Wound at one-week postoperative follow-up.

Tissue histopathology returned positive for zygomycosis with necrotic adipose tissue and suppurative inflammation (Figure 3). At one-week follow-up, the wound was clean and granulating (Figure 4), and she was systemically well. Given her clinical improvement, after discussion with the Infectious Diseases team, she was not given antifungal treatment. Final blood and tissue cultures returned negative. Panfungal PCR did not detect fungal infection, which is likely due to specimen limitations (only four-week-old formalin-fixed tissue was available for PCR analysis). The wound had healed at a four-week postoperative review.

## 3. Discussion

This case highlights some important practice points regarding cutaneous mucormycosis infection. Firstly, cutaneous mucormycosis typically follows direct inoculation, such as after trauma, burns, or surgery. Previous outbreaks of cutaneous mucormycosis have been also associated with natural disasters and combat-related injuries [3]. *Apophysomyces*, *Saksenaea* species, and *Lichtheimia corymbifera* species are relatively frequently reported causative agents in trauma-related mucormycosis [3]. However, reports of mucormycosis infection have been also linked to minor skin trauma, including insect bites, intravenous cannulation, and subcutaneous injection [4, 5]. As seen in our case, cutaneous mucormycosis can be associated with minor breeches of the skin barrier. This should be carefully elicited on history and exam, in addition to other risk factors for infection. A review of 176 patients with cutaneous mucormycosis found independent risk factors of female sex (OR, 2.27; 95% CI, 1.46–3.55), prior surgery (OR, 5.40; 95% CI, 1.84–15.86), and HIV infection (OR, 2.62; 95% CI, 1.01–6.79) [4]. While typically associated with immunosuppression (such as diabetes, HIV/AIDS, cancer, and, in recent times, COVID-19 infection [6]), a significant proportion of patients with cutaneous infection are immunocompetent [4, 7–10]. Other risk factors for infection include iron overload and treatment with iron chelators, which promote fungal growth [1].

Secondly, clinicians should maintain a high degree of clinical suspicion for cutaneous mucormycosis to aid with timely diagnosis. Wound cultures are often falsely negative, and there are no rapid diagnostic serology/PCR tests currently available [1]. Maintaining a high index of suspicion will prompt surgeons to pursue tissue biopsy, which will show characteristic broad, ribbonlike, pauciseptate, or nonseptate fungal hyphae [1, 11]. Fresh tissue specimens can also be sent for panfungal PCR (results to be interpreted with caution as PCR can be overly sensitive to contaminant nonpathogenic fungi). Fresh tissue is preferred over paraffin-embedded tissue for molecular-based methods because formalin damages DNA [10].

Thirdly, the hallmark of cutaneous mucormycosis infection is tissue necrosis, due to angioinvasion and intravascular thrombosis. Complications include deep tissue spread, necrotizing fasciitis, and disseminated disease. Thus, the mainstay of treatment is aggressive (and potentially disfiguring) surgical debridement, as supported by the 2019 consensus guidelines for the diagnosis and management of mucormycosis [10]. Repeated surgical exploration may be required for gangrenous disease [1]. Surgical reconstruction can be considered once clinical improvement of the debrided wound is confirmed [12]. IV amphotericin B is used for empirical antifungal therapy, with newer azoles (posaconazole and isavuconazole) used for stepdown or salvage therapy. Based on the observational data, nondisseminated cutaneous mucormycosis treated with aggressive surgical debridement and adjunct antifungal therapy carries a favourable prognosis [4, 13].

To conclude, we report a rare case of cutaneous mucormycosis in an immunocompetent patient who had no predisposing risk factors, other than the recent innocuous skin trauma. The cornerstones of management remain prompt aggressive surgical debridement to prevent dissemination to deeper tissues, antifungal agents, and optimisation of underlying immunosuppression.

## Data Availability

No data were used to support this study.
